# Shaping a knowable event or embracing a mysterious journey: A mixed methods study on palliative care clinician views on voluntary assisted dying

**DOI:** 10.1017/S1478951525100655

**Published:** 2025-09-03

**Authors:** Graham Llewellyn Grove, Melanie R. Lovell, Phyllis Butow, Ian Hughes, Megan Best

**Affiliations:** 1 Specialist Palliative Care, Gold Coast Hospital and Health Service, Southport, QLD, Australia; 2 School of Medicine, The University of Sydney, Camperdown, NSW, Australia; 3 Department of Palliative Care, HammondCare, Greenwich Hospital, Greenwich, NSW, Australia; 4 Northern Clinical School, School of Medicine, The University of Sydney, Camperdown, NSW, Australia; 5 Psycho-Oncology Co-operative Research Group (PoCoG), School of Psychology, The University of Sydney, Camperdown, NSW, Australia; 6 Research Office, Gold Coast University Hospital, Southport, QLD, Australia; 7 Institute for Ethics and Society, The University of Notre Dame Australia, Broadway, NSW, Australia

**Keywords:** Voluntary assisted dying, euthanasia, physician-assisted suicide, palliative care, clinician views

## Abstract

**Objectives:**

This study explored Australian palliative care clinicians’ perspectives on the legalization of voluntary assisted dying (VAD), aiming to identify variables associated with clinicians’ views and understand challenges of its implementation.

**Methods:**

An online survey exploring support for legalization of VAD was sent to palliative care clinicians in Queensland and New South Wales and followed up with semi-structured interviews. Support was categorized as positive, uncertain, or negative. An ordinal logistic regression model was used to identify variables independently predictive of euthanasia support. Interviews were analyzed using grounded theory to understand key concepts shaping views on VAD.

**Results:**

Of 142 respondents, 53% supported, 10% were uncertain, and 37% opposed legalizing euthanasia for terminal illness with severe symptoms. Support was lower for patients with chronic illness (34%), severe disability (24%), depression (9%), severe dementia (5%), and for any adult with capacity (4%). The model showed lower support among doctors than nurses (38% vs. 69%, *p* = 0.0001), those in New South Wales compared with Queensland (44% vs. 69%, *p* = 0.0002), the highly religious versus least religious (24% vs. 79%, *p* = 0.00002), those politically conservative versus progressive (39% vs. 60%, *p* = 0.04), and those with more healthcare experience (*p* = 0.03). Seventeen interviews revealed 2 distinct groups: one focused on the event of death and the need to relieve suffering, providing comfort in the final moments; the second on the journey of dying and the possibility of discovering peace despite suffering. Those in the first group supported legal VAD, while those in the second opposed it. Despite opposing views, compassion was a unifying foundation common to both groups.

**Significance of results:**

There are 2 fundamentally different orientations toward VAD among palliative care clinicians, which will likely contribute to tensions within teams. Acknowledging that both perspectives are rooted in compassion may provide a constructive basis for navigating disagreements and supporting team cohesion.

## Introduction

Euthanasia and physician-assisted suicide (PAS) are topics that often evoke strong emotions and diverse opinions among the public and healthcare professionals. Euthanasia refers to the administration of a substance by a healthcare professional to intentionally end a person’s life, frequently in the context of a voluntary request from a patient with a terminal illness causing unrelieved suffering (Güth et al. 2023). PAS involves the prescription of a substance for self-administration with the same intent. Voluntary assisted dying (VAD) and medical assistance in dying are more recent legal terms that can encompass either or both of these constructs.

Historically, VAD has been illegal; however, several jurisdictions have legalized VAD under specific circumstances in recent decades (Fontalis et al. 2018; Grove et al. 2025). When this study was conducted, VAD remained illegal in both New South Wales (NSW) and Queensland (QLD). However, throughout 2022, with impending legalization of VAD in QLD, healthcare professionals across the state were actively engaged in learning about and preparing for VAD implementation, set for 1 January 2023. In contrast, legalization of VAD remained uncertain in NSW in early 2022, although it was under active debate in the state parliament. By mid-2022, however, a bill had been passed to allow for legal VAD in NSW to commence in late 2023.

Legalization of VAD has, inevitably, had direct impacts on clinicians involved in end-of-life care. Therefore, understanding the views of these clinicians is important to inform the broader discussions surrounding the legalization of VAD and its implementation in clinical practice. Notably, clinicians who care for patients at the end of life, particularly those in palliative care, have generally been less supportive of legalizing VAD than other clinicians with previous studies indicating that palliative care doctors are about half as likely as non-palliative care doctors to support legal VAD (Grove et al. 2025; Marini et al. 2006; Seale 2009). Previous studies have also shown doctors to show less support for legal VAD than nurses (Glebocka et al. 2013; Grove et al. 2025; Zenz et al. 2015).

In QLD and NSW, with populations of approximately 5.5 million (Queensland Government 2024) and 8.2 million, respectively (NSW Government 2022), palliative care services are delivered through a mix of private and public providers, operating in a decentralized system. Without a centralized registry of palliative care practitioners, the exact number of clinicians is unknown. A 2022 review of the QLD palliative care workforce estimated there were 66 full-time equivalent specialist palliative care doctors and 140 full-time equivalent specialist palliative care nurses working in the state (Queensland Government 2022).

This study aimed to explore the perspectives on VAD held by palliative care doctors and nurses in QLD and NSW, in the lead-up to the implementation of legal VAD in QLD, but not in NSW. In particular, the study sought to examine potential associations between support for legal VAD and role, level of experience, religious beliefs, and the region of clinical practice, as well as the deeper question of why clinicians hold their beliefs about VAD and how these perspectives are formed. To address the aim, a mixed methods approach was applied to enable analysis of links between quantifiable trends and underlying values shaping clinician attitudes, with the reporting of the methods and findings guided by principles of *Good Reporting of a Mixed Methods Study* (O’Cathain et al. 2008)

## Methods

### Participants

Participants for the survey were recruited through QLD’s and NSW’s professional palliative care organizations. Eligibility criteria, explicitly noted in the survey introduction, included: working in the field of palliative care as a doctor or nurse. This study received ethics approval from the University of Sydney (2022/462).

### Data collection

An online survey (Supplementary Table 1) and questions for a subsequent semi-structured interview (Supplementary Table 2) were developed based on a review of academic literature regarding factors influencing euthanasia (E) and PAS beliefs, collectively referred to as VAD. In QLD, the online survey was distributed via email to the members of the state’s Palliative Care Medical Directors Group, with a request to circulate it to all doctors and nurses working in or affiliated with their services. In NSW, distribution occurred through the Sydney Institute of Palliative Medicine, a large network of clinicians with an interest in palliative care. The survey collected demographic data and views of palliative care doctors and nurses on VAD in QLD and NSW in 2022, prior to legalization of VAD. Participants were asked whether they supported euthanasia and PAS under certain circumstances, opposed it under all circumstances, or were uncertain about their stance.

Following the survey, a subset of respondents was contacted via SMS and invited to participate in a semi-structured interview via Microsoft Teams. Purposive sampling was used to allow a heterogenous sample. Participants were asked about their work experiences, worldviews, understandings of VAD, perspectives on its ethics, and predictions of its impacts on society, healthcare, and their personal lives. All interviews were conducted by G.L.G., a physician with qualifications in palliative medicine and theology. Interviews were recorded and transcribed.

### Analysis

Support for VAD in relation to demographic, professional, religious, and political factors collated from the online survey was analyzed using Stata/BE 19.0 (College Station, TX, USA). Participants’ support for euthanasia and PAS was categorized as “Yes” (support under certain circumstances), “No” (opposition under all circumstances), and “Uncertain” with proportions and 95% confidence intervals (CIs) reported for each category. In addition to this general question, respondents were asked if they supported VAD in specific scenarios with responses recorded as “Yes” or “No” and corresponding 95% CIs calculated.

The influence of state (QLD or NSW), sex, age, profession (nurse or doctor), experience (years in healthcare and palliative care), job satisfaction, importance of religion or spirituality, and political leaning (conservative or progressive) on VAD support was also investigated. For each predictor, the proportions supporting VAD or not, 95% CIs, and an overall *p*-value were calculated (Fisher’s exact test). Ordinal logistic regression was employed to construct a model that identified which of these variables independently influenced support for VAD. Each variable was initially assessed individually and considered for inclusion in a multivariate model if *p* < 0.1. Variables were retained in the multivariable model if *p* < 0.05 for that variable. To aid interpretation, the independent effect of each variable in the model was presented as predicted proportions of support for each level of the predictor variable along with its overall *p*-values. Potential collinearity between predictor variables was assessed by observation of Spearman’s rank correlations and calculation of the variance inflation factor.

Qualitative analysis was undertaken concurrently with the interviews using a grounded theory approach based on principles outlined in Charmaz (2006) and incorporating elements of the framework method for analysis (Gale et al. 2013). The survey data from individual interviewees were linked to their corresponding verbatim transcripts, which were then coded inductively by G.L.G., M.C.B., M.R.L., and P.B. using line-by-line analysis. A coding tree was developed and data collection was continued until saturation (Glaser and Strauss 1967). G.L.G., M.C.B., and M.R.L. then convened to examine relationships between categories and integrate disparate codes into concepts and themes that described perspectives on VAD.

The varied backgrounds of the researchers (palliative medicine, psycho-oncology, ethics, and biostatistics) and heterogeneous beliefs regarding legalization of VAD facilitated reflexivity.

## Results

### Quantitative results

A total of 142 participants responded. Aged between 23 and 80 years, nearly two-thirds of participants were from NSW, half were doctors, and the majority were female (Table 1). Among females, 54% were nurses and 42% doctors; among males, 14% were nurses and 82% doctors. The distribution of medical to nursing staff, and religious to non-religious participants was similar between states; however, QLD participants had fewer years of palliative care experience, with 49% having less than 10 years compared to 37% in NSW.

**Table 1.** Survey participant characteristics

		Number of participants (% of total)
All respondents		142 (100%)
State	NSW	87 (61)
	QLD	49 (35)
	Other	6 (4)
Sex	Female	114 (80)
	Male	28 (20)
	Other	0 (0)
	Prefer not to say	0 (0)
Profession	Doctor	71 (50)
	Nurse (including nurse practitioners)	65 (46)
	Nurses (excluding nurse practitioners)	58 (41)
	Nurse practitioner	7 (5)
	Other	6 (4)
Years in health care	<10 years	25 (18)
	10−20 years	42 (29)
	>20 years	74 (53)
Years in palliative care	<10 years	57 (40)
	10 − 20 years	47 (33)
	>20 years	36 (25)
Job satisfaction	Unsatisfied (very or somewhat)	31 (22)
	Neither satisfied nor unsatisfied	6 (4)
	Satisfied (very or somewhat)	105 (74)
Importance of religious or spiritual beliefs	Extremely important	41 (29)
	Somewhat important	45 (32)
	Not particularly important	38 (27)
	Definitely not important	18 (13)
Political leaning	Conservative-leaning	17 (12)
	Progressive-leaning	69 (49)
	Neither	56 (39)

Overall, 54% (95% CI 45% to 62%) supported legalizing euthanasia under some circumstances, 37% (95% CI 29% to 45%) opposed it, and 10% (95% CI 6% to 16%) were uncertain. Support for PAS was similar (53% [95% CI 45% to 61%]), but with lower opposition (32% [95% CI 25% to 40%]) and higher uncertainty (15% [95% CI 10% to 22%]). The highest level of euthanasia support (52%) was for patients with symptomatic terminal illnesses (Table 2). Support decreased to 32% for asymptomatic terminally ill patients, 34% for symptomatic chronically ill patients, 24% for those with permanent disabilities, and 36% for those with severe dementia with an advance health directive requesting euthanasia. The lowest support was for people with mental health conditions (9%), advanced dementia without an advance directive (5%), and any competent adult seeking to end their life (4%).

**Table 2.** Support for VAD based on access criteria

Age	Key criterion	Sub-criterion	Euthanasia support	95% CIs
Adult	Terminal illness (prognosis of months or less)	Symptoms	52%	44% to 60%
		Without symptoms	32%	25% to 40%
		Dying and drowsy, in the last weeks of life (without capacity)	11%	7% to 17%
	Chronic illness	Symptoms	34%	27% to 42%
		Without symptoms	8%	4% to 13%
	Disability	Poor quality of life	24%	18% to 32%
	Dementia	Mild (with capacity)	15%	10% to 22%
		Severe without capacity (but with advance health directive)	36%	28% to 44%
		Severe without capacity (but on family request)	5%	2% to 10%
	Depression	Poor quality of life	9%	5% to 15%
	Any reason	Has capacity	4%	2% to 9%
Teenager	Terminal	Unbearable symptoms	37%	30% to 46%
	Disability	Unbearable quality of life	15%	10% to 22%
Neonate	Terminal	Unbearable symptoms (assessed by the parents)	30%	23% to 38%
	Disability	Unbearable (assessed by the parents)	4%	2% to 9%

Ordinal logistic regression identified that religion, political leaning, years in healthcare, role (doctor or nurse), and state (QLD or NSW) independently influenced support for euthanasia. Variables that did not appear to independently influence support for euthanasia were age, sex, years in palliative care, and job satisfaction. A Spearman rank correlation matrix and variance inflation factor analysis confirmed that, although some of these predictive variables were related, their individual influence could still be separated. The adjusted predicted percentages for each category of support for each predictor variable in the model are shown in Table 3. Doctors were less likely to support euthanasia than nurses (38% versus 69%), NSW clinicians were less supportive than Queenslanders (44% versus 69%), the longer time spent working in health care, the less supportive a clinician was, the most religious were less supportive than the least religious (24% versus 79%), and the politically conservative were less supportive than the politically progressive (39% versus 60%).

**Table 3.** Expected percentage support for and opposition to legalized euthanasia across key independent predictor variables, derived from ordinal logistic regression

Variable		Support	Uncertain	Opposition	*p*-value
State	QLD	69% [60% to 79%]	8% [4% to 13%]	22% [14% to 31%]	0.0002
	NSW	44% [36% to 52%]	11% [6% to 17%]	45% [37% to 53%]	
Role	Doctor	38% [28% to 48%]	12% [6% to 18%]	50% [41% to 60%]	0.0001
	Nurse	69% [60% to 77%]	9% [4% to 13%]	22% [15% to 30%]	
Religion in personal life	No importance	79% [57% to 99%]	8% [2% to 14%]	13% [0% to 28%]	0.00002
	Minor importance	60% [47% to 73%]	12% [6% to 18%]	29% [17% to 40%]	
	Moderate importance	62% [51% to 73%]	11% [6% to 17%]	27% [17% to 37%]	
	Extremely important	24% [12% to 36%]	11% [6% to 17%]	65% [51% to 78%]	
Political leaning	Conservative	39% [20% to 59%]	10% [5% to 15%]	50% [31% to 69%]	0.04
	Neither conservative nor progressive	47% [37% to 57%]	10% [5% to 15%]	43% [33% to 53%]	
	Progressive	60% [53% to 68%]	10% [5% to 15%]	30% [23% to 38%]	
Years in healthcare	5 years	66% [53 to 79%]	9% (4% to 13%]	25% [14 to 36%]	0.03
	15 years	57% [50 to 64%]	10% [5% to 15%]	33% [26 to 40%]	
	25 years	47% [39 to 56%]			

Figures in square brackets represent 95% confidence intervals.

The survey identified concerns regarding the legalization of VAD, with 34% (95% CI 27% to 42%) believing that VAD would negatively impact their work environment. In contrast, 18% (95% CI 12% to 25%) anticipated an overall positive impact, 19% (95% CI 13% to 26%) predicted a neutral impact, and 29% reported uncertainty about VAD’s potential impact.

### Qualitative results

Seventeen in-depth interviews were conducted, 10 with nurses (2 nurse practitioners) and 7 with doctors. Ten participants worked in urban palliative care services and 7 in rural settings. Ten supported legalization of VAD (2 doctors and 8 nurses) and 7 did not (5 doctors and 2 nurses). Interview duration ranged from 45 to 90 minutes.

Consistent with the survey findings, interview participants expressed clear positions for or against VAD legalization with 7 opposed and 10 supporting. Three major themes connected with this were identified: (1) understandings of dying and death; (2) perspectives on suffering; and (3) identity rooted in compassion (Table 4).

**Table 4.** Themes underlying formation of VAD beliefs

	Supporters of VAD (*n* = 10)	Opponents of VAD (*n* = 7)
**Key demographics**	2 doctors, 2 nurse practitioners, 6 nurses	5 doctors, 2 nurses
	3 considered religion extremely important (30%) (1 Catholic, 1 “progressive Christian,” 1 “agnostic” but “spiritual”)	3 considered religion extremely important (43%) (2 Baptist, 1 Pentecostal)
		3 considered religion somewhat important (43%)
	3 considered religion somewhat important (30%)	1 considered religion not important (14%)
	4 considered religion not important (40%)	
**Major themes**		
Understandings of dying and death	Predominantly discussed death as an event and the moments surrounding it	Predominant discussed dying as a journey and the time leading up to death as potentially positive
	9 supporters of VAD (90%) spoke about death in this way	6 opponents of VAD (86%) spoke about dying in this way
Perspectives on suffering	Intolerable suffering is unacceptable when it can be alleviated through a patient’s choice	Peace and meaning can be found whilst suffering, thus outweighing its harm
	All 10 supporters of VAD spoke in these terms (100%)	6 opponents of VAD (86%) spoke about suffering in this way
Identity rooted in compassion	Compassion is especially shown in facilitating a peaceful death free from suffering	Compassion is especially shown in supporting a patient to search for peace and connection despite suffering (rather than indicating their suffering renders their life hopeless or meaningless)
	9 supporters of VAD (90%) spoke about compassion in this way	
		5 opponents of VAD (71%) spoke about compassion in this way
**Minor themes**	7 supporters (70%) discussed *autonomy*; however, 3 emphasized this but only in the context of suffering (30%)	5 opponents (71%) discussed *prohibition against killing* 4 opponents (57%) discussed *protecting the vulnerable* 2 opponents (29%) discussed *difficulties with prognostication*

#### Understandings of dying and death

Beginning the interview with open questions about work experiences and VAD perspectives led many participants to reflect on death and dying. A prominent theme was that, whilst some participants believed other clinicians – particularly outside of palliative care – viewed death as failure, they themselves did not. One participant, speaking of oncologists, said that:

It’s almost like, for most doctors, death is failure – patients are not supposed to die because that means I’ve failed them. (Interviewee 14)

For participants, death was seen as inevitable, and so failure was linked to a bad death, not death itself. Success was seen as facilitating a “good death.” Helping someone die well was central to participants’ sense of purpose in their work:

The whole reason I’ve stayed in palliative care for so long is that, there’s a chance of dying well or a chance of them dying badly and if by doing good clinically we can make people die well, then that’s a real achievement. I think that’s always been my drive for the job. (Interviewee 1)

Some participants acknowledged that the idea of a good death is subjective, recognizing it was impossible to have a universally agreed understanding of dying well. Reflecting on the recent death of a patient, one participant noted the individual perspectives of the patient’s family:

He probably would have said she had a good death, whereas the sister said she had a bad death. (Interviewee 14)

As participants explored the complexities of dying and death, a subgroup emerged that emphasized an ongoing process over time of caring for terminally ill patients. Using words like “journey” and “process” and phrases such as “dying takes time,” this group focused on the period leading up to death, rather than the event of death itself. This emphasis of journey was common among those who opposed VAD and some participants spoke about palliative care in terms of living:

We’re about making people live as well as they can until they die. (Interviewee 8)

In this context of journey, some participants described a potential beauty and reward despite the challenges faced by dying individuals, warning against overlooking this potential:

Attempting to learn from the challenges and beauty and the rewards that the dying process provides is often overlooked. (Interviewee 2)

When speaking of “uncertainty” and “mystery,” some participants described how the dying journey allowed for profound and beautiful moments with family, and how life goals could still be achieved:

There is a journey that any patient goes through when they have a terminal diagnosis. And I think there are moments that are beautiful moments that can be shared together with family. (Interviewee 2)

In contrast, a second subgroup emerged where the focus of discussion was on the event of death itself. These participants spoke about providing good care at the time of death, using words like “peaceful,” “good,” and “comfortable,” alongside phrases such as “not lingering,” “not waiting,” and “it was quick.” Several participants described the beauty found in a peaceful death. One participant, for instance, spoke of a distressed patient with a fungating tumor who settled with midazolam and sedation:

It was a beautiful death.” (Interviewee 4)

The cohort that centered discussion on the moments of death, mostly favored the option of legal VAD for patients.

Thus, 2 distinguishable groups were identified: those who emphasized a longer journey of dying, appreciating potential beauty in this process; those who focused on the event of death itself, valuing the peacefulness that might be fostered in the moments that surrounded it.

#### Perspectives on suffering

Like a “good death,” many participants also highlighted the subjective nature of suffering, noting the difficulty in defining and measuring it objectively:

Who assesses suffering? Suffering is totally subjective, and it’s hard for a healthcare provider to put a value on your suffering. (Interviewee 16)

Participants described suffering as more than physical pain, highlighting emotional and spiritual dimensions. Reflecting on her training, one palliative medicine specialist emphasized the role of a multiprofessional team in addressing suffering:

I saw such complex suffering be managed really well because we had a pastoral care service, volunteers, everything. (Interviewee 2)

There was strong confidence in many participants in the ability of palliative care teams to alleviate suffering, with participants expressing pride in this:

We actually prevent suffering! (Interviewee 6)

However, although participants found fulfillment in this aspect of their work, many acknowledged limitations:

I don’t believe that palliative care answers every single piece of the puzzle of suffering or every single element of suffering in our patients. (Interviewee 2)

This recognition extended to both physical and non-physical unresolved suffering:

We can’t get rid of everybody’s existential suffering, or, indeed everybody’s physical pain. (Interviewee 15)

For some, this limitation led to a sense of sadness, and even helplessness:

There are some symptoms, including existential distress that we just can’t fix. (Interviewee 12)

As with the divergence between those focused on the event of death versus the dying journey, 2 contrasting perspectives on suffering also emerged – one centered on removing all suffering; the other on finding meaning in its midst. Accordingly, some participants saw suffering as fundamentally unacceptable. This shaped their professional goal to do everything possible to eliminate suffering. Describing the distress of witnessing such patients, some participants likened patients to animals in similar situations:

We can’t always fix physical symptoms and existential distress which leaves people to live in a state of suffering. I wouldn’t let my dog live like that. My dog would be at the vet, being put down in its own best interest, however hard that is for me. Yet we leave people living in states that we wouldn’t leave an animal living in. (Interviewee 1)

Participants discussed the impact of suffering, not just on patients, but also on their loved ones:

I’ve seen some really terrible deaths where we have just not been able to get symptoms managed. Families have suffered and the patient has suffered. I think that in some cases, maybe voluntary assisted dying is a way that people don’t have to suffer. (Interviewee 13)

These participants leaned toward a worldview where no one should need to endure ongoing suffering. In this perspective, individuals had a right to a way out of such suffering. This group, who also framed the discussion around death as an event, generally supported legal access to VAD:

And sometimes we can’t ease their suffering. To my mind, VAD is for them. VAD is for people suffering, and it isn’t just physical, it’s the emotional and mental suffering too. (Interviewee 3)

In contrast, other participants viewed suffering as an unavoidable, mysterious aspect of human existence, inherent to the frailty of being human. Some suggested that suffering could, sometimes, have meaning:

Suffering is part of having a human existence. And part of journey is learning how to sit with that. Choosing to end one’s life is not a solution. (Interviewee 11)

Some participants described society as increasingly uncomfortable with suffering, becoming reluctant to engage in discussions about it:

Even a little bit of suffering, people are uncomfortable with it… Death and suffering have really been taken out of society. (Interviewee 6)

They worried that a growing discomfort with suffering in our society might diminish the value of human life for the frail, sick, or dying. One participant even contrasted the difference between animals and humans:

I think humans are different to animals… Growing old and becoming frail is part of life. Suffering is part of life and sometimes there is some value in suffering, sometimes. I think that the older and sicker people fall, that they can become less valued. (Interviewee 5)

Although this group of participants expressed a deep commitment to alleviating suffering, they also saw their role as guiding patients and families to find meaning, value, and even hope in the midst of suffering:

Life is valuable to the last day, despite what people think. So, I feel we are losing opportunity to look after our patients and show compassion if we give them VAD… Some people [who are dying] have very fulfilling experiences, meeting family and meeting their goals. (Interviewee 6)

Several participants took an especially broad view of suffering, suggesting that VAD will not end it, recognizing that suffering continues in family and friends as grief endures beyond death itself. For some family members and loved ones, this grief may be compounded by lingering questions about whether there might have been another way without VAD, which may deepen distress rather than bring closure:

Suffering is going to exist in some form. VAD means an end to whatever suffering they [the patients] are experiencing. But there are implications beyond that in terms of suffering of the family and the broader community. (Interviewee 11)

#### Identity rooted in compassion

Despite these 2 distinct groups with opposing perspectives on VAD, a unifying theme of compassion for the suffering was apparent. Indeed, compassion was a central factor in shaping participants’ understanding of their identities as palliative care clinicians. They viewed it as an intrinsic part of their professional and personal selves. One nurse reflected on this when describing a personal tragedy:

And you know, it’s like they say in the Bible that often you get growth from tragedy. It sounds horrendous, but it’s true. Unfortunately, it’s so true. I just think my compassion grew. (Interviewee 3)

The influence of this compassion on VAD perspectives was tied to participants’ views on dying and suffering. Support for legal VAD was linked to those who perceived relentless and unrelievable suffering as the ultimate harm – almost never acceptable.

No one should have to suffer. And in saying that, why would you prevent someone making a choice to take their own life if that’s their personal choice? (Interviewee 10)

These participants saw themselves as playing a critical role in ensuring the event of death was peaceful, without pain and suffering:

I want to help ease people’s suffering toward the end of life. And sometimes we can’t ease it. To my mind, VAD is for them. VAD is for people suffering. (Interviewee 3)

Their concern extended beyond physical symptoms to include emotional distress in response to loss, both current and anticipated. One doctor, reflecting on her grandmother’s course post-stroke, suggested the most compassionate course of action would have been VAD.

And she was moved into a nursing home… So, she requested to die, asking that we would just kill her and I think that would have been a kind outcome for her in that situation. That was an emotional experience. (Interviewee 10)

Like those supporting legal VAD, participants who opposed it were also driven by compassion. However, their compassion led them to reject VAD:

I feel like we are losing the opportunity to look after our own and show compassion, if we actually give them VAD. (Interviewee 6)

These participants believed VAD deprived patients of opportunities to find meaning and peace, stripping them of precious time with their loved ones. One participant, reflecting on a family friend with metastatic cancer, spoke candidly about this:

I think, for me, maybe it’s because I have small children, but I feel like if he were to go down that path [of VAD], it would be reducing the time he has with his children. (Interviewee 16)

Another participant described a more profound, spiritual loss that could occur with an early death precipitated by VAD:

I think euthanasia would prevent patients of that journey, of that appreciation and understanding of that mystery of dying. (Interviewee 2)

Some participants expressed sadness that legal VAD might lead patients to question their inherent value:

I feel sad because I think some people will be persuaded just by society that VAD is good… Older and sick people will feel that when they’re given VAD as an option, that they’re less valued and that they’re a burden on the health system. (Interviewee 5)

Ultimately, these participants did not view VAD as a compassionate response to suffering. One participant even suggested that the very idea of a solution to suffering was an illusion:

The idea that we can have that [a solution to suffering] capitulates to the myth that we can control everything, that everything can be on our own terms. That’s just an illusion. (Interviewee 11)

## Discussion

This study explored the perspectives of palliative care doctors and nurses in QLD and NSW on VAD legalization through a survey and interviews. Most clinicians had strong opinions, which is unsurprising in palliative care clinicians who regularly care for dying patients and frequently encounter questions about VAD.

A slight majority of palliative care clinicians supported VAD legalization for symptomatic, terminally ill patients, a finding not anticipated based on previous research (Grove et al. 2025). Factors such as shifting societal values and understandings of medical ethics may contribute to this higher-than-expected support. However, without the criteria of terminal illness and severe symptoms, majority support was not observed. This aligns with the legal criteria for VAD in Australia, raising the question: Did knowledge of impending legislation with these criteria influence clinician support or, alternatively, was the legislation shaped to reflect the views of healthcare professionals involved in end-of-life care?

While more palliative care clinicians supported VAD legalization than opposed it for patients with symptomatic, terminal illness, almost none supported it in cases of mental illness, dementia without capacity, or those “tired of life.” Despite this, some countries, including the Netherlands and Canada, have either legalized or are considering legalizing VAD in these situations (Kouwenhoven et al. 2018; van Veen et al. 2022). Thus, understanding why clinicians oppose VAD in these contexts is valuable.

Our interviews suggest that both support for and opposition to VAD among palliative care clinicians is fundamentally linked to compassion. Support is borne out of a compassionate desire to give patients death without suffering, a finding aligning with previous research (Digby et al. 2020; Hewitt et al. 2023). This explains the stronger support for VAD in cases of symptomatic chronic illness (as opposed to chronic illnesses without physical suffering). Likewise, support is stronger for patients with terminal illnesses, even in the absence of physical symptoms, than for non-terminal illnesses, as VAD is a means of ending the intense psychological and existential distress in individuals who know they are dying. In cases where significant suffering is not evident – such as in chronic, minimally symptomatic conditions or elderly individuals who feel their lives are complete – clinicians generally oppose legal access to VAD. Thus, neither our interviews nor our survey indicates that autonomy, or the right to self-determination, is a primary driver for support of VAD legalization, contrasting with research suggesting otherwise (Hewitt et al. 2023; Sandham et al. 2022). Instead, our data indicated clinicians primarily support legalization of VAD in situations where the access criteria align with what they see as a compassionate response.

Our research also found that doctors are generally less supportive of VAD than nurses, consistent with existing literature (Grove et al. 2025). If compassion guides VAD beliefs, differences in nurses’ and doctors’ roles may shape how this compassion is expressed (Malenfant et al. 2022). Nurses typically spend more time with patients in their final days, closely witnessing pain or distress up close, yet may have less ability to address such suffering directly, particularly when access to a doctor or nurse practitioner for pharmacological intervention is limited. A compassionate response toward patients, their families, and even oneself may therefore involve a strong desire to alleviate the observed suffering, which could strengthen their support for VAD. In contrast, doctors usually have less prolonged contact with patients and may thus be less emotionally impacted by their suffering. Additionally, doctors are aware that they may carry a unique burden in that they may be expected to administer VAD. The weight of this responsibility of directly ending a patient’s life may place a profound emotional burden on doctors. Consequently, their compassion for themselves in connection with their professional identity may be contributing to their higher levels of opposition to VAD.

Our study, consistent with prior research, found that religious individuals are far less supportive of legal VAD than their non-religious counterparts (Grove et al. 2025). When considering the 2 groups that emerged in our interviews – those focused on the event of death versus those emphasizing the longer journey of dying – the possibility is raised that the religious divide links to broader understandings of life and faith as journey (Cole 1984; Wiebe 2022) where individuals make discoveries, seek forgiveness, and find peace (Exline et al. 2012; Renz et al. 2020). For clinicians of faith, compassion may mean helping patients find meaning, peace, forgiveness, and reconciliation. Moreover, their concepts of peace and suffering may be more eternally focused when compared to their less religious counterparts. Among the least religious populations, where a person’s experience and existence are not understood to extend beyond this life, peace may be viewed as attainable only in this life, within the present moments of suffering. Their compassionate response aligns with a consequentialist ethical perspective, where the kindest response is offering control to end suffering by VAD. This stands in contrast to the more religious, whose worldview often involves a sense of mystery with the need to relinquish control, allowing a journey of dying to unfold along an unknown path. From this perspective, intervening to cause death will interrupt this journey. Furthermore, death does not necessarily relieve suffering (Grove et al. 2022). Thus, the compassionate response is to assist the dying person to find peace, a peace that may extend beyond this life.

One aspect of our study that remains difficult to explain is the lower support for VAD among palliative care clinicians in NSW. Given the relatively small sample size of 142, this may be due to chance. It does however raise questions about whether the societal and political contexts around the impending implementation of legal VAD in QLD (but not NSW) influenced clinician perspectives. In the months leading up to VAD in QLD, which was also when this survey was conducted, significant education and local unit discussions took place. These discussions may have led to greater acceptance of VAD, even though its practice had not yet commenced. Alternatively, differences in training or leadership, or differences in availability of palliative care support, could explain the discrepancy between states.

One limitation of this study is the small sample size, reducing the statistical power of results. This limitation is further compounded by the non-random nature of the sample. Furthermore, the survey did not capture whether clinicians practiced in urban or rural settings, limiting the ability to determine whether this aspect of practice context influenced the findings. Similarly, in the qualitative interviews, selection bias may have impacted results. While potential interviewer bias was identified and attempts made to minimize its impact, it is possible that the interviewer’s perspective was, at times, perceived by participants, potentially influencing their responses.

## Conclusion

This study demonstrates that, prior to the legalization of VAD in QLD and NSW, palliative care clinicians generally held well-defined ethical positions on the issue. Approximately half expressed clear support for legal VAD, while slightly fewer were firmly opposed. These divergent views were rooted in differing conceptual orientations toward death and suffering. Despite the strength of these opposing perspectives, a consistent and unifying theme of compassion emerged across both groups. As VAD becomes legally available, recognizing that compassion underpins the positions of both proponents and opponents may support constructive dialogue and help clinicians maintain respectful and collaborative relationships within palliative care teams.

## Supporting information

10.1017/S1478951525100655.sm001 Grove et al. supplementary material 1 Grove et al. supplementary material

10.1017/S1478951525100655.sm002 Grove et al. supplementary material 2 Grove et al. supplementary material

## Data Availability

Requests for data and materials will be considered through discussion with the University of Sydney Ethics Committee that approved this research project.

## Supplementary material

The supplementary material for this article can be found at https://doi.org/10.1017/S1478951525100655.
