# Supplementary material for: Shaping a knowable event or embracing a mysterious journey: A mixed methods study on palliative care clinician views on voluntary assisted dying
Source: Palliat Support Care. 2025 Sep 3;23:e153. doi: 10.1017/S1478951525100655 (PMC13166625; doi:10.1017/S1478951525100655)
Supplement: Grove et al. supplementary material 1 — Grove et al. supplementary material [file S1478951525100655sup001.docx]

Supplementary Table 1

| This survey should take between 5 and 10 minutes to complete.  Thank you for taking part in the following survey of the views of Australian specialist palliative care and oncology clinicians regarding euthanasia and assisted suicide.  You will be asked to provide your email so that a follow-up survey can be sent to you in 9-12 months, however all data collected will be de-identified prior to any analysis by researchers.  On submission, you are consenting to involvement in this online survey.  For the purposes of this survey, unless otherwise specified assume the following: - The patient is an adult - Euthanasia is defined as the injection of a medication with the primary intention of ending a patient's life painlessly at the request of a patient deemed to have capacity - Assisted suicide is defined as the prescription (at the request of the patient) of an oral medication at a dose that would cause a painless death so that the patient is able to take the medication at the time of their choosing to precipitate their death - Voluntary assisted dying refers to either euthanasia or assisted suicide as defined above  Further details about this research can be found in the attached Patient Information Statement.  Finally, please consider forwarding this on to your colleague clinicians working in specialist palliative care and oncology. |
| --- |
| In general, do you support **legal euthanasia**under certain conditions?  (Single choice only)  *Yes / No / Uncertain* |
| If yes, under what circumstances do you think **euthanasia should be legal**?  Terminal illness (death expected within months) + severe symptoms (e.g. pain)  Terminal illness (death expected within months) regardless of symptoms  Chronic illness (life expectancy of potentially many years) + severe symptoms (e.g. pain)  Chronic illness (life expectancy of potentially many years) regardless of symptoms  Chronic disability impacting quality of life  Depression or another mental illness impacting quality of life  Any adult with capacity who wishes euthanasia regardless of the reason  Early, mild dementia in a person deemed to still have capacity  Dementia where capacity is no longer present but where a written advance health directive requests euthanasia  Dementia where capacity is not present but the closest family member or enduring power of attorney requests euthanasia  A drowsy or confused person assessed as dying by the treating medical team as being in the last few days of life and the closest family member requests euthanasia  A neonate or young child with a terminal illness and severe symptoms and the parent or guardian requests euthanasia  A neonate or young child with a disability and the parent or guardian requests euthanasia  A teenager with a terminal illness and severe symptoms who requests euthanasia  A teenager with a severe disability who requests euthanasia  (Single choice answer per question)  *Yes / No* |
| In general, do you support **legal assisted suicide**under certain conditions?  (Single choice only)  *Yes / No / Uncertain* |
| Terminal illness (death expected within months) + severe symptoms (e.g. pain)  Terminal illness (death expected within months) regardless of symptoms  Chronic illness (life expectancy of potentially many years) + severe symptoms (e.g. pain)  Chronic illness (life expectancy of potentially many years) regardless of symptoms  Chronic disability impacting quality of life  Depression or another mental illness impacting quality of life  Any adult with capacity who wishes euthanasia regardless of the reason  Early, mild dementia in a person deemed to still have capacity  A teenager with a terminal illness and severe symptoms who requests euthanasia  A teenager with a severe disability who requests euthanasia  (Single choice answer per question)  *Yes / No* |
| Please select the primary field you currently work in.  (Single option only)  Palliative care / Oncology / Something else |
| Where do you currently work?  (One option only)  *Queensland / New South Wales / Somewhere else* |
| If you answered "somewhere else" above, please specify where you work |
| Which best describes your role and qualifications?  (Single choice only)  *Medical Consultant / Advanced Trainee / Nurse Practitioner / Clinical Nurse Consultant / Clinical Nurse / Registered Nurse / Enrolled Nurse / Assistant in Nursing / Something else* |
| How many years have you worked as a healthcare clinician? |
| How many years have you been working in the field of palliative care? |
| What is your sex?  (Single choice only)  *Male / Female / Non-binary / Prefer not to say* |
| What is your age? |
| What are the key components of your work?  (Multiple choices allowed)  Inpatient care / Outpatient care / Community care / Research / Administration / Education |
| How satisfied are you in your work life?  (Single choice only)  Very unsatisfied / Somewhat satisfied / Neither satisfied nor dissatisfied / Somewhat satisfied / Very satisfied |
| Are you thinking about leaving work in your current discipline and field in the next 5 years?  (Single choice only)  No / Yes - thinking about retiring / Yes - thinking about retiring / Yes - thinking about retiring |
| If yes to the question above, can you provide more details? |
| Would you be willing to be involved in the provision of euthanasia if legal?  (Single choice only)  Yes / No |
| Would you be willing to be involved in the provision of assisted suicide if legal?  (Single choice only)  Yes / No |
| Do you believe that, overall, euthanasia, assisted suicide and voluntary assisted dying will have either a negative or positive impact on your work?  (Single choice only)  Generally a positive impact / No significant impact / Generally a negative impact / Uncertain |
| Please explain your answer above |
| In terms of your political values, which best describes your views  (Single choice only)  Leaning towards the conservative side of politics / Leaning towards the progressive side of politics / Neither well describes me |
| How would you describe your ethnic and/or cultural background? |
| What religion and/or denomination do you identify as? (e.g. Christian, Roman Catholic, Sunni Muslim, non-religious, atheist) |
| At a day to day level, how important are your religious beliefs or spirituality to you?  (Single choice only)  Extremely important / Somewhat important / Not particularly important / Definitely not important |
| Would you consent to an online or in-person interview with the lead researcher to further explore your views for qualitative research purposes?  (Single choice only)  Yes / No |
| Supplemental Table 1: Online survey |
