# Supplementary material for: Shaping a knowable event or embracing a mysterious journey: A mixed methods study on palliative care clinician views on voluntary assisted dying
Source: Palliat Support Care. 2025 Sep 3;23:e153. doi: 10.1017/S1478951525100655 (PMC13166625; doi:10.1017/S1478951525100655)
Supplement: Grove et al. supplementary material 2 — Grove et al. supplementary material [file S1478951525100655sup002.docx]

Supplementary Table 2

| Tell me about your work, your job, over the last year. |
| --- |
| How do you define euthanasia, assisted suicide and voluntary assisted dying. |
| Tell me about your thoughts on euthanasia, assisted suicide and voluntary assisted dying. |
| How have you come to form these thoughts and beliefs? |
| Do you think you will be involved in the provision of VAD when it becomes legal?   - If so, what aspects of voluntary assisted dying might you participate in? - If not, how will approach not being involved? |
| How do you think legal VAD would impact you on a personal level, and your work in your specific job?   - Are there any challenges you anticipate? - Are there any emotional, psychological or spiritual issues you have thought about? |
| How do you think legal VAD will impact our health-care system? |
| How do you think legal VAD will impact society?  In both the short-term? And in the longer term? |
| Tell me your thoughts about legal VAD criteria? |
| Do you think you would be likely to seek VAD for yourself in certain circumstances? |
| What about close family members – do you think you would help them seek VAD and be supportive of this for them in certain circumstances? |
| We’re coming to the end of the interview now. Do you have any final issues you would like to talk about? |
| ***Supplementary Table 2:*** *Prepared interview questions asked of each participant. In addition, the interviewer asked further questions to probe answers and follow tangents.* |
